# Genome Sequence of *Rickettsia hoogstraalii*, a Geographically Widely Distributed Tick-Associated Bacterium

**DOI:** 10.1128/genomeA.01171-14

**Published:** 2014-11-06

**Authors:** Erwin Sentausa, Khalid El Karkouri, Thi-Tien Nguyen, Aurélia Caputo, Didier Raoult, Pierre-Edouard Fournier

**Affiliations:** Aix-Marseille Université, URMITE, UM63, CNRS 7278, IRD 198, INSERM U1095, Faculte de Medecine, Marseille, France

## Abstract

*Rickettsia hoogstraalii* is a tick-associated member of the spotted fever group rickettsiae that is geographically widely distributed. We report here the draft genome of *R. hoogstraalii* strain Croatica^T^ (=DSM 22243 = UTMB 00003), which was isolated from *Haemaphysalis sulcata* ticks collected in Croatia.

## GENOME ANNOUNCEMENT

The genus *Rickettsia* consists of Gram-negative obligate intracellular bacteria that are associated with arthropods. The spotted fever group of this genus is made of species causing tick-borne rickettsioses, which are among the oldest known vector-borne diseases. *Rickettsia hoogstraalii* is a spotted fever group of rickettsiae that was described in 2010 (1). This species is closely related to *Rickettsia felis*. Although its pathogenesis in vertebrate hosts is unknown, *R. hoogstraalii* causes a cytopathic effect in Vero, CCE3, and ISE6 cells. Originally isolated in 2006 from *Haemaphysalis sulcata* ticks from Croatia (2) and *Carios capensis* ticks from the United States (3), it has been detected in other tick species in different parts of the world, including Japan (4), Spain (5), Cyprus (6), Ethiopia (7), Turkey (8), and the western Indian Ocean (9). Here, we briefly describe the genome sequencing of *R. hoogstraalii* strain Croatica^T^ (= DSM 22243^T^ = UTMB 00003^T^).

The genome was sequenced using MiSeq technology (Illumina, San Diego, CA, USA) with a mate-pair strategy. SPAdes-3.1.0 (10) was used to perform a *de novo* assembly of the reads, and the best assembly with a *k*-mer value of 127 was chosen for annotation. Potential coding sequences (CDSs) were predicted using AMIGene (11), and the assignment of protein functions was performed by searching against the RickBase (12), GenBank, and Pfam (13) databases using BLASTp (14), while ribosomal RNAs, tRNAs, and other RNAs were identified using BLASTn, tRNAscanSE version 1.21 (15), and RNAmmer 1.2 (16). Orthologous genes between the chromosomes of *R. hoogstraalii* and *R. felis* strain URRWXCal2 (GenBank accession no. NC_007109.1) were identified using OrthoMCL (17), with a BLASTp *E* value cutoff of 1 × 10^-5^ and the default MCL inflation parameter of 1.5.

The draft genome of *R. hoogstraalii* Croatica^T^ consists of two contigs of 1,444,049 nucleotides and 40,763 nucleotides, respectively, with an average genome coverage of 326-fold and a G+C content of 32.38%. The shorter contig is a putative plasmid with an identity match of 83% (35% coverage, *E* value 0.0) to Plasmid01 from *Rickettsia australis* strain Cutlack (accession no. CP003339.1) when aligned using BLASTn. The chromosome contains 1,824 CDSs and, like other rickettsiae, 3 noncontiguous rRNAs (5S, 16S, and 23S rRNA), 33 tRNAs, and 3 other RNAs. In addition, the plasmid contains 70 CDSs but no RNAs.

Compared to the *R. felis* chromosome, several genes are lacking in *R. hoogstraalii*, including genes encoding a putative esterase and a putative hydrolase/acyltransferase of the α/β hydrolase superfamily, a toxin of an toxin-antitoxin system that contains a PIN domain for nucleic acid binding (*vapC2*), a site-specific DNA methylase, a superfamily I DNA and RNA helicase, a sugar kinase from the ribokinase family, three guanosine polyphosphate pyrophosphohydrolases/synthetases (*spoT5*, *spoT8*, and *spoT10*), the cell surface antigen Sca11, a major facilitator superfamily (MFS)-type permease (*proP*), an alkylated DNA repair protein, the DNA-damage-inducible protein J (*relB2*), the MnhF subunit of a multisubunit Na+/H+ antiporter, a glutamine amidotransferase-like protein, and a penicillin acylase.

### Nucleotide sequence accession numbers.

The genome and plasmid sequences have been deposited in DDBJ/EMBL/GenBank under accession numbers CCXM01000001 and CCXM01000002, respectively.
